# Demonstrated Progress and Future Promise of Chronic Disease Data Modernization

**DOI:** 10.5888/pcd21.240396

**Published:** 2024-10-31

**Authors:** Kathryn Turner, Katherine H. Hohman

**Affiliations:** 1 Idaho Department of Health and Welfare, Boise, Idaho; 2 National Association of Chronic Disease Directors, Decatur, Georgia

## Introduction

Public health surveillance is defined as the continuous and systematic collection, analysis, and interpretation of data and is the cornerstone of public health action (1). The US has historically underfunded public health surveillance systems, leading to a fragmented and outdated data infrastructure (2,3). Historic “feast or famine” and disease-specific funding strategies resulted in a siloed, archaic, and inflexible public health data ecosystem.

Recent investments in public health data modernization (4) offer the promise of a future integrated data ecosystem that allows public health practitioners, scientists, academics, and medical professionals to better understand and prevent chronic disease in the US. In some jurisdictions, implementation of innovative demonstration and pilot projects is laying the groundwork for broader adoption of modernized surveillance practice. Collaboration and coordination across state, tribal, local, and territorial (STLT) public health agencies and other partners are critically important for systemic improvements in chronic disease surveillance, improved health equity, and a reduced burden of chronic disease (5).

## A Shared Vision but Different Challenges

Protecting and improving population health is the goal of both infectious and chronic disease surveillance (6). However, unlike the systems that STLT agencies have for surveilling acute infectious diseases, many of these agencies do not have policy levers available to collect granular data for chronic diseases and thus rely on secondary data sources for timely surveillance. Lack of governance and comprehensive data exchange policies limits access to the data necessary for public health decision-making and evaluation of public health interventions (7). A modernized data ecosystem will require identifying opportunities for improvements in the policy and legal frameworks that govern data sharing for chronic disease surveillance.

The data challenges in chronic disease prevention are considerable and include ensuring data granularity at a level that allows practitioners to evaluate community-level progress, compare across communities and over time, and measure progress toward health equity and improvements in the social determinants of health such as reducing adverse childhood events (8). The articles in this collection highlight significant advancements in chronic disease surveillance, emphasizing the importance of collaboration and coordination among partners. They describe solutions that predominantly use electronic health record (EHR) systems data, demonstrating the potential of EHRs in modernizing surveillance. These articles also underscore the challenges of chronic disease surveillance, such as data quality validation and the need for consensus approaches to defining chronic conditions. Finally, these articles discuss future considerations, including comprehensive strategies to address health equity and social determinants of health in the context of chronic disease prevention and control.

## Collaboration and Coordination Are Foundational for Data Modernization

The need for collaboration and coordination in modernizing chronic disease surveillance is paramount and is an important thread woven through each of the articles in this collection. Collaboration is necessary to address the challenges related to data quality and representativeness and the fragmentation of health care systems (9). Wiltz and colleagues (10) summarize the impact of the CDC Federal Data Strategy and the key roles public health, research, and other communities play in data modernization. By working together, public health agencies, health care systems, and research networks can address these challenges and leverage EHR data for more accurate and timely surveillance of chronic diseases. Collaborative efforts can also promote the development of sustainable surveillance tools, data integration across silos, and the inclusion of diverse populations, ultimately leading to better data for decision-making in chronic disease management and prevention.

The article by Barth and colleagues (11) describing Michigan’s efforts to create a chronic disease registry acknowledges that longstanding relationships with health systems and collaboration with the statewide health information exchange (HIE) are key to their ongoing efforts. Kader and associates (12) emphasize the importance of collaboration among community-based organizations, academic researchers, health care providers, and government officials to improve surveillance for chronic disease in New York State as part of the IDEAL (Innovations in Data Equity for All Laboratory) initiative.

Ghildayal and coauthors (13) and Jackson and colleagues (14) describe leveraging the National Patient-Centered Clinical Research Network (PCORnet) as a foundation for their work in modernizing surveillance practice. This network of participating health care institutions and partners works with a coordinating center to make data available to improve health outcomes. Through this collaborative infrastructure, the authors highlight how networks such as PCORnet facilitate the use of EHR data for public health surveillance and promotion of health equity.

## Integrating Secondary Data Use in Data Modernization: Electronic Health Records

Many of the articles in this collection focus on leveraging EHR systems data to improve chronic disease surveillance. Traditional population-based surveillance systems are not designed to provide timely, local, and comprehensive data and can be supplemented with EHR data for more robust chronic disease surveillance. Carney and coauthors (15) describe the use of these data as an important priority area for data modernization planning at the Centers for Disease Control and Prevention (CDC). However, this secondary data source is based on data collected not for population or public heath purposes but for patient-centered individual medical care documentation and decision-making. The public health approach to using these data requires ongoing investment, research, and continued evaluation.

Ghildayal and associates, in studying how EHR data contained in PCORnet can supplement traditional surveillance practice, conclude that these data offer advantages such as timeliness, availability for large populations, and the ability to capture longitudinal data and are a source of objective data on disease prevalence, incidence, and control (11). PCORnet added functionality to make zip code–level analysis easier to conduct as well as a modular program to generate population health statistics across conditions. The authors leveraged these EHR data to study 2 chronic disease surveillance case studies on atrial fibrillation and cirrhosis. Jackson and coauthors (14) examine trends in the use of preventive services and new diagnoses of chronic diseases among more than 30 million US adults by using PCORnet. Their analysis revealed a decline in both use of preventive services and new chronic disease diagnoses during and after the COVID-19 pandemic, highlighting the need for public health intervention to address barriers to care and ensure equitable access to preventive services.

Barth and colleagues (11) describe the development of Michigan’s Chronic Disease Registry Linking Electronic Health Record Data (CHRONICLE), a near-real-time disease monitoring system designed to leverage EHR data and existing HIE infrastructure for chronic disease surveillance. The authors conclude that the experience in Michigan demonstrates the value of leveraging EHR data and HIE infrastructure for innovative public health surveillance approaches to address the rising burden and costs associated with chronic diseases.

Using EHR data in public health surveillance offers significant promise in the ability to describe the burden of chronic disease in the US, assess progress in minimizing its impact on communities, and evaluate interventions at levels of granularity often not available by using traditional surveillance methods, both at the geographic level and among demographic groups. Integrating EHR data into routine chronic disease surveillance would improve the epidemiology of these diseases by allowing analysis of granular population-level assessments, risk factors for disease, social determinants of health, and progress toward health equity goals.

## Standardization, Quality, and Validation of Data Sources for Modernization

Among the articles in this collection, notable challenges to modernizing surveillance include a discussion of the limitations of using secondary data sources for chronic disease surveillance. Ghildayal and colleagues (13) note that although EHRs are a rich source of data, they have limitations, such as incomplete data, lack of representativeness, and challenges with data quality and integration across different health systems. Wiltz and coauthors (10) identify good data hygiene practices to consider in data modernization plans, including data management and organization; code, software, and statistical processing practices for manipulation and analysis; and collaboration and offer a checklist that can be used when planning data modernization for chronic disease surveillance.

Barth and coauthors (11), in evaluating Michigan’s CHRONICLE, conclude that the usefulness of EHR data depends on establishing data quality standards to enable broader use of clinical data in public health practice. The authors monitored incoming hospital ADT (admit, discharge, transfer) messages to understand the completeness and quality of the data, noting variations in coding formats and missing data fields but also recognizing the potential of the data for chronic disease surveillance. They emphasize that creating clear data quality standards specific to public health use of EHR data remains a critical gap that requires dedicated efforts at the national, state, and regional levels.

The Multi-State EHR-Based Network for Disease Surveillance (MENDS) project provides insights into both data validation and the effect of analytic decisions on surveillance estimates. In their first article in this collection, Hohman and coauthors (16) detail the validation process implemented by MENDS to identify and resolve data quality issues that could affect chronic disease prevalence estimates that use EHR data. The validation process uncovered various challenges, such as missing data, incomplete patient records, and issues with data processing and standardization that underscore the importance of independent data validation before using raw EHR data for public health surveillance. The authors provide a roadmap of data validation methodology and highlight the value and importance of data validation to improve the accuracy of surveillance estimates that use EHR data. The lessons learned can be broadly applied to other EHR-based surveillance efforts.

In their second article, Hohman and colleagues (17) describe the impact of analytic decisions on prevalence estimates of hypertension identified from EHR data. They demonstrate how different analytic decisions in defining both numerators and denominators significantly affect EHR-based estimates of chronic disease prevalence and control. The lack of a consensus definition for hypertension exacerbates the challenge of calculating prevalence. However, the results of this study have implications that extend beyond the MENDS project to inform clinical and public health efforts in conducting surveillance of hypertension prevalence and control in other data systems and populations.

The IDEAL initiative, undertaken to improve the collection, analysis, and communication of race and ethnicity data in New York State, underscores the need to address shortcomings of race and ethnicity data collection within secondary data systems used in public health practice. Kader and colleagues (12) emphasize the importance of ongoing community engagement, patient-centered approaches, and a commitment to racial equity throughout the data lifecycle to ensure data accurately represent the diversity of communities.

## Chronic Disease Data Modernization: Future Considerations

Two key themes emerge from this collection of articles. First, investments in data improvement must adopt an evergreen approach, emphasizing the need for an ongoing, iterative system life cycle that requires continuous investment, evaluation, validation, and enhancement. Ghildayal and colleagues (13) conclude that using EHR data for public health surveillance requires strategic funding models and support at federal, state, and local levels to ensure EHRs remain a sustainable tool for surveillance. This approach applies broadly to any efforts aimed at improving chronic disease surveillance.

Second, continued and *expanded* partnerships are critical to success. The articles in this collection clearly illustrate the need for public health collaboration with health care providers, academia, private and public technology developers, and private partners with expertise in data technology and use. Data modernization requires not only technology innovation but also the development of standardized methodologies and analytical approaches. For instance, the Fast Healthcare Interoperability Resources (FHIR) standard provides a framework for exchanging electronic health information, facilitating interoperability between different health systems (18). By adopting FHIR and similar standards, data can be more easily shared and integrated, enhancing the capacity for chronic disease surveillance.

Chronic diseases must be included and prioritized in the national Public Health Data Strategy (19) and related initiatives. Ensuring chronic disease surveillance goals are integrated into funding strategies and technical assistance efforts and are used as capacity-building programs is essential for the advancement of technology, data tools, analysis methods, and workforce capabilities. Although progress has been made in modernizing surveillance for acute and infectious diseases in the aftermath of the COVID-19 pandemic, more needs to be done to address chronic diseases, the leading causes of death and disability in the US. Solutions that benefit acute and infectious disease categories should benefit chronic disease surveillance and epidemiology. Collaboration with colleagues working in infectious disease surveillance will be critical to achieving health equity goals across these public health domains.

Opportunities for future advancements include leveraging the Trusted Exchange Framework and Common Agreement (TEFCA), a public–private US network connecting HIEs since late 2023 (20). Future efforts should ensure that chronic disease data use cases are integrated into that agreement and similar infrastructures.

Equally important is leveraging the US Core Data for Interoperability (USCDI) process to standardize the core data elements needed for chronic disease surveillance (21). The USCDI’s structured and evolving framework ensures that critical data, including social determinants of health and patient demographic information, are uniformly collected and exchanged across EHR systems. Incorporating chronic disease data elements into future versions of the USCDI will enhance data interoperability, consistency, and accuracy for public health efforts.

In addition, the North Star Architecture, an initiative within CDC, is a cloud-based service model to benefit STLT public health departments by using shared tools, applications, and a flexible architecture to integrate data from multiple sources, data streams, and shared data for public health action (22). Adding data sources over time for chronic disease epidemiology and prevention would address many of the challenges identified by the authors of the articles in this collection related to data quality, validation, and standardization.

However, a modern technology architecture alone will not resolve all the challenges. The public health workforce must adapt to the use of new technology and new approaches to data collection, management, provisioning, analysis, and use. Epidemiologists, data scientists, engineers, and others involved need the skills to fully leverage the modern data ecosystem. Ensuring ongoing investments in capacity and skill building, as well as fostering partnerships with academic and private partners, will bolster public health’s ability to meet the goals of chronic disease prevention — reduced disease burden and impact, increased health equity, and reduced health care costs. Only by advancing chronic disease data modernization can we equip public health to meet not just today’s challenges but those of the future.
